# Tongue exercise ameliorates structural and functional upper airway deficits in a rodent model of hypoglossal motor neuron loss

**DOI:** 10.3389/fneur.2024.1441529

**Published:** 2024-09-04

**Authors:** Amy N. Keilholz, Ishan Pathak, Catherine L. Smith, Kate L. Osman, Lauren Smith, Grace Oti, Mojgan Golzy, Lixin Ma, Teresa E. Lever, Nicole L. Nichols

**Affiliations:** ^1^Department of Biomedical Sciences, College of Veterinary Medicine, University of Missouri, Columbia, MO, United States; ^2^Department of Veterinary Pathobiology, College of Veterinary Medicine, University of Missouri, Columbia, MO, United States; ^3^Research Division, Biomolecular Imaging Center, Harry S. Truman Memorial Veterans’ Hospital, Columbia, MO, United States; ^4^Department of Physics and Astronomy, College of Arts and Science, University of Missouri, Columbia, MO, United States; ^5^Department of Otolaryngology-Head and Neck Surgery, School of Medicine, University of Missouri, Columbia, MO, United States; ^6^Biostatistics Unit, Department of Family and Community Medicine, University of Missouri, Columbia, MO, United States; ^7^Department of Radiology, School of Medicine, University of Missouri, Columbia, MO, United States; ^8^Department of Medical Pharmacology and Physiology, School of Medicine, University of Missouri, Columbia, MO, United States; ^9^Dalton Cardiovascular Research Center, University of Missouri, Columbia, MO, United States

**Keywords:** motor neuron disease (MND), breathing, respiration, dysphagia, degeneration, rat model

## Abstract

**Introduction:**

Tongue weakness and atrophy can lead to deficits in the vital functions of breathing and swallowing in patients with motor neuron diseases (MNDs; e.g., amyotrophic lateral sclerosis (ALS) and pseudobulbar palsy), often resulting in aspiration pneumonia, respiratory failure, and death. Available treatments for patients with MNDs are largely palliative; thus, there is a critical need for therapies targeting preservation of upper airway function and suggesting a role for tongue exercise in patients with MNDs. Here, we leveraged our inducible rodent model of hypoglossal (XII) motor neuron degeneration to investigate the effects of a strength endurance tongue exercise program on upper airway structure and function. Our model was created through intralingual injection of cholera toxin B conjugated to saporin (CTB-SAP) into the genioglossus muscle of the tongue to induce targeted death of XII motor neurons.

**Methods:**

Rats in this study were allocated to 4 experimental groups that received intralingual injection of either CTB-SAP or unconjugated CTB + SAP (i.e., control) +/- tongue exercise. Following tongue exercise exposure, we evaluated the effect on respiratory function (via plethysmography), macrostructure [via magnetic resonance imaging (MRI) of the upper airway and tongue], and ultrafine structure [via ex vivo magnetic resonance spectroscopy (MRS) of the tongue] with a focus on lipid profiles.

**Results:**

Results showed that sham exercise-treated CTB-SAP rats have evidence of upper airway restriction (i.e., reduced airflow) and structural changes present in the upper airway (i.e., airway compression) when compared to CTB-SAP + exercise rats and control rats +/- tongue exercise, which was ameliorated with tongue exercise. Additionally, CTB-SAP + sham exercise rats have evidence of increased lipid expression in the tongue consistent with previously observed tongue hypertrophy when compared to CTB-SAP + exercise rats or control rats +/- tongue exercise.

**Conclusion:**

These findings provide further evidence that a strength endurance tongue exercise program may be a viable therapeutic treatment option in patients with XII motor neuron degeneration in MNDs such as ALS. Future directions will focus on investigating the underlying mechanism responsible for tongue exercise-induced plasticity in the hypoglossal-tongue axis, particularly inflammatory associated factors such as BDNF.

## Introduction

Movement of the tongue is important for the vital functions of breathing and swallowing. The genioglossus muscle of the tongue is the primary active dilator of the upper airway during normoxia, as anterior movement of the genioglossus at the level of the epiglottis during inspiration helps maintain airway patency (1). Upper airway dysfunction in motor neuron diseases (MNDs; e.g., amyotrophic lateral sclerosis (ALS), spinobulbar muscular atrophy/Kennedy’s disease and progressive bulbar palsy) involve complex processes leading to the loss of motor neurons that innervate the muscles of mastication, facial muscles, and/or muscles of the palate and pharynx/larynx (e.g., cranial nerves V, VII and IX/X respectively) (2). However, upper airway dysfunction in MNDs is predominantly thought to be attributed to degeneration of motor neurons (MNs) in the hypoglossal (XII) axis, including XII lower motor neurons (LMNs) that innervate the genioglossus muscle causing extensive tongue weakness, leading to impaired breathing and swallowing functions (3–6). Breathing and swallowing require reciprocal roles of the tongue during swallowing, and impaired control and coordination of these opposing behaviors can result in aspiration pneumonia, respiratory failure, and ultimately death (2, 7–9). Few studies have investigated therapeutic strategies to preserve breathing and/or swallowing function in MNDs. Thus, existing treatments are palliative and do not significantly improve functional outcomes and quality of life, which highlights the critical need for therapies aimed at preserving upper airway function. Studies focused on early detection have identified impaired tongue strength as an independent prognostic indicator of shorter survival in ALS (10, 11), suggesting that tongue exercise may play an essential therapeutic role in patients with MND.

Upper airway dysfunction in ALS models is difficult to study since the rate which hypoglossal motor neuron death occurs cannot be controlled, and degeneration is not limited to the hypoglossal nucleus. Thus, to study this, we utilized our validated rodent model with targeted loss of XII motor neurons, which provide the sole motor innervation to the tongue via the hypoglossal nerve. This model was created by intralingual injection of cholera toxin B conjugated to saporin (CTB-SAP) into the genioglossus muscle of the tongue base, which is transported retrogradely to XII motor neurons. Upon entering the XII LMN cell bodies, CTB-SAP dissociates and SAP is free to bind to ribosomes and inhibit protein synthetic machinery, resulting in apoptotic cell death (12, 13). Our previous investigations with this model revealed degenerative changes in the XII nerve (i.e., denervation atrophy) and genioglossus muscle [i.e., myofiber atrophy; (14)]. Additionally, *in vivo* magnetic resonance imaging (MRI) in this model revealed evidence of macrostructural changes in the brainstem (e.g., a trend for 4^th^ ventricle enlargement) and tongue (e.g., significantly increased tongue volume and thickness, and marked hyperintensity of the tongue) consistent with potential muscle fiber inflammation, fatty replacement (possibly due to increased lipid concentration) of atrophied muscle fibers, and/or edema which were somewhat mitigated via tongue exercise in CTB-SAP rats (15).

The goal of the current study was to investigate whether tongue weakness and hypertrophy observed in this inducible model would result in evidence of decreased airflow (i.e., decreased peak inspiratory flow and increased inspiratory time) consistent with upper airway restriction, upper airway degenerative changes (i.e., compressed airway lumen), and ultrafine structural changes in the tongue (e.g., increased lipid profiles) that could be prevented by implementing targeted tongue exercise. To test this, we utilized our previously defined high-repetition/low-resistance (i.e., strength endurance) exercise paradigm designed for muscle growth (15), which consists of two non-consecutive overnight training sessions (Days 4 and 6 after intralingual injections).

We hypothesized that: (1) sham exercise-treated CTB-SAP rats would develop evidence of decreased airflow consistent with upper airway resistance (e.g., decreased peak inspiratory flow and mean inspiratory flow, and increased inspiratory time) during normoxic conditions at endline; (2) upper airway restriction in CTB-SAP rats during normoxia would be mitigated by tongue exercise; (3) lipid profiles in the tongue of CTB-SAP sham-exercise treated rats would be increased, consistent with fatty deposition of atrophied muscle fibers; (4) macrostructural and ultrafine structural degenerative changes in the tongue would be mitigated by tongue exercise in CTB-SAP rats; and (5) sham exercise-treated CTB-SAP rats would have degenerative changes in their upper airway (i.e., compressed airway lumen) consistent with evidence of upper airway restriction. These complementary studies may provide translationally relevant insight for early clinical indication of XII LMN degeneration. Overall, these findings help us understand the potential benefits of tongue exercise as a therapeutic strategy in patients with MND to preserve life-sustaining upper airway function and structure.

## Materials and methods

### Animals

Experiments were conducted on adult (3–4 months old) male Sprague Dawley rats (Envigo Colony 208a; Indianapolis, IN, United States). Rats were pair-housed in standard vivarium conditions (ambient temperature 20–26° C, humidity 30–70%, and standard 12:12 light:dark cycle). Animals had access to a standard commercial pelleted diet and water *ad libitum*, except during experimental testing (described below). Daily health monitoring and routine surveillance for common rodent illnesses were performed by veterinary staff. All experimental procedures were approved by our Institutional Animal Care and Use Committee and conducted in accordance with the Guide for the Care and Use of Laboratory Animals within our USDA-licensed and AAALAC-accredited academic institution.

### Experimental procedures

Rats (*N* = 50) were randomly allocated to four experimental groups to study the effects of tongue resistance exercise on tongue-related structure and function. All rats received intralingual injection of either unconjugated CTB + SAP (i.e., control) or conjugated CTB-SAP, followed by exposure to either tongue exercise (i.e., treatment) or sham exercise (i.e., sham treatment), as described in detail below. Before tongue injections and after exercise/sham exercise exposure (i.e., 8 days after tongue injection), rats underwent behavioral testing to evaluate respiratory function via whole body plethysmography. At the study endpoint (i.e., 9 days after tongue injection), a subset of 13 rats underwent *in vivo* MRI of the upper airway to investigate corresponding structural changes under normoxic conditions that may correlate with behavioral findings. Additionally, *ex vivo* MRI and MR spectroscopy were performed on the tongues of a subset of rats (*N* = 24) to study macrostructural and ultrafine structural changes in the tongue, which included studying the metabolites of the tongue with a focus on lipid profiles. This study endpoint is the same as our previous studies with this model (14, 16), which was determined based on pilot data showing that other time points either did not result in dysphagia (4 days post tongue injection) or resulted in severe dysphagia (11–14 days post tongue injection) compromising animal welfare. All rats were euthanized on Day 9 using American Veterinary Medical Association (AVMA) approved methods. Specifically, rats were euthanized via exsanguination under deep isoflurane anesthesia by transcardial perfusion with cold PBS/saline (0.9% NaCl in 0.01 M sodium phosphate buffer, pH 7.0) followed by paraformaldehyde (in 0.1 M sodium phosphate buffer, pH 7.4) for collection of tissue for future histological analysis. In all cases, death was confirmed via bilateral pneumothorax where the chest cavity was opened, and lack of respiration and heartbeat were confirmed.

#### Intralingual injections to create an inducible rat model of selective hypoglossal motor neuron degeneration

Our rat model was created as previously described (14, 16). In brief, rats were anesthetized via 5% isoflurane in an induction chamber and immobilized in ear bars in the supine position on a custom-built tilt table to stabilize the head during tongue injections. For the remainder of the procedure, isoflurane (2–3% via 1–1.5 L/min oxygen) was delivered via nose cone to extinguish hindlimb and jaw reflexes. The jaw was gently held open by a custom-built weighted pulley-mechanism looped around the mandibular incisors for unobstructed access to the tongue. Under light guidance (LED Stereotactic Light #59290, Stoelting; Wood Dale, IL, United States), fine forceps were used to gently grasp and lift the tongue for visualization of the frenulum, which was the anatomical landmark for targeted injection into the midline genioglossus muscle in the tongue base. Each rat received a single “control” injection (20 μg CTB + 25 μg SAP; unconjugated CTB + SAP) or CTB-SAP injection (25 μg of CTB conjugated to SAP) using a 50 μl Luer tip syringe (Microliter #705, Hamilton; Reno, NV, United States) and 26-gauge Luer lock needle (26G 3/8 Becton, Dickinson and Company, Franklin Lakes, NJ, United States). The needle was angled at 45-degrees during insertion into the midpoint of the frenulum, with half of the bolus delivered at ~8 mm depth (i.e., near maximum needle insertion) and the remainder at ~4 mm (i.e., half the needle insertion depth) during needle retraction. All injections were performed by the same investigator for replicability of results. Rats were recovered from anesthesia (typically within 10 min after induction) and closely monitored for several hours to ensure resumption of food and water intake before being returned to standard vivarium conditions and daily health monitoring.

#### Whole-body plethysmography to assess respiratory function

Respiratory function was evaluated in CTB-SAP rats and controls rats (CTB + SAP) +/− tongue exercise via plethysmography by placing the rats in a whole-body plethysmograph (Data Sciences International/Harvard Bioscience, Holliston, MA) and altering the mixture of inspired gases (gas concentrations controlled by a gas mixer, CWe, Inc., Ardmore, PA). Testing was conducted on all rats prior to tongue injections (i.e., baseline) and on day 8 following tongue injections (i.e., endline). Baseline respiratory function was established by first exposing the animals to 30 min of normoxia. All rats were then challenged by exposing them to 5 min. of a hypoxic + hypercapnic gas mixture (10.5% O_2_, 7% CO_2_, balanced N_2_; maximum chemoreceptor stimulation, max). A pressure calibration signal, ambient pressures, and chamber pressures were utilized for automated calculation of breath-by-breath respiratory parameters [frequency (f), inspiratory time (TI), expiratory time (TE), tidal volume (VT), minute ventilation (V.E), peak inspiratory flow (PIF) and peak expiratory flow (PEF)] at 10-s intervals using FinePointe Software (Data Sciences International/Harvard Bioscience, Holliston, MA). VT, V.E, and mean inspiratory flow (VT/TI) were normalized to body weight (per 100 g). Data were rejected in rare instances of pressure fluctuations caused by gross body movements. Additionally, the apnea detection function within FinePointe software was used to identify an additional outcome measure, total number of apneas, that were defined as the absence of at least two inspirations (i.e., a pause in breathing 2x the normalized breath duration threshold). The automatically detected apneas were manually reviewed to verify accuracy and exclude rare instances of automated event detection errors, for example, if two shallow breaths were detected as an apnea rather than two individual breaths.

#### Tongue exercise paradigm

Prior to tongue injections, force-lickometer testing was performed to determine the baseline maximum voluntary lick force (MVLF) and corresponding personalized tongue exercise intensity level for each rat (15). Testing entailed individually enclosing rats in a custom lickometer chamber (clear polycarbonate, 25 cm long × 8 cm wide × 15 cm high) positioned on a custom vertical lift platform within a modified force-lickometer system (Force Lickometer for Rat, Med Associates; Fairfax, VT, United States). A custom lickometer spout (i.e., a funnel with an adjustable force, double ball-bearing spout) was filled with a 30% sucrose solution to motivate participation, and the lick-force threshold to obtain liquid from the spout was set to ≤4 grams to mimic the negligible lick-force requirement of our vivarium’s standard double ball-bearing waterspouts. Additionally, a webcam (V-u0018, Logitech; Newark, CA, United States) was positioned inside the lickometer system for continuous close-up visualization of the spout. As each rat drank, tongue contact against the spout was captured (via PowerLab data acquisition device; ADInstruments; Colorado Springs, CO, United States) for real-time visual display and recording (via LabChart software; ADInstruments) of tongue force in grams (g) in synchrony with the webcam video stream. Each rat was recorded for approximately 5 min to obtain multiple drinking bouts for MVLF analysis. Offline, the best representative 3–5 drinking bouts (i.e., continuous drinking with visible tongue contact against the spout) per rat were manually identified for automated quantification (via LabChart) of peak-to-peak amplitude (g) for individual licks. The 10 highest lick-force values across the multiple bouts were averaged to obtain each rat’s MVLF, which was used to determine the individualized exercise program for each rat as described below.

All rats participated in our established strength endurance tongue exercise paradigm on Day 4 and Day 6 post-tongue injection (15), which necessitated single housing for the remainder of the study to ensure a personalized medicine approach. On both days, a custom exercise spout (i.e., resisto-spout) containing 30% sucrose solution was placed in the home cage of individually housed rats for 12 h overnight (i.e., 8:00 PM–8:00 AM), coinciding with peak activity (including food/water consumption) in these nocturnal rodents. The spout was customized with a manually adjustable tension spring mechanism with a force range of ~2–50 g to accommodate sham exercise (≤4 g) and exercise (~20–35 g, based on unpublished pilot testing) conditions. Each resisto-spout force setting was manually calibrated immediately prior to use with one of two analog tension force meters: low range (0–10 g; model GD-1, Jonard Tools, Elmsford, NY United State) for sham exercise or high range (0–50 g; model GD-5, Jonard Tools) for exercise.

For rats in the two exercise groups (i.e., control + exercise and CTB-SAP + exercise), the resisto-spout was set to 50% greater than baseline MVLF (i.e., 50% > MVLF) throughout the overnight exercise period. For example, a rat with a MVLF of 20 g would have the resisto-spout set to 30 g during exercise. For the two sham exercise groups (i.e., control + sham exercise and CTB-SAP + sham exercise), the resisto-spouts were set to ≤4 grams, consistent with the negligible force setting used during force-lickometer testing, as conducted previously. Rats had free access to standard food pellets and enrichment materials during exercise/sham exercise training in the home cage; thus, the only cage-level difference was the substitution of the resisto-spout bottle (containing 30% sucrose solution) for the standard vivarium water bottle.

#### *Ex vivo* magnetic resonance imaging and spectroscopy of the tongue

*Ex vivo* magnetic resonance imaging (MRI) and magnetic resonance spectroscopy (MRS) was performed on a subset of rat tongues (*N* = 19) using a 7 T Bruker AVANCE III BioSpec MRI scanner (Bruker BioSpin Inc., Billerica, MA) equipped with a mouse brain CryoProbe (Bruker Biospin). On day 9 following tongue injections, rats were transcardially perfused with 4% paraformaldehyde in 0.1 M phosphate-buffered saline (PBS, pH~7.4) and the tongues were harvested. *Ex vivo* tongue T2-weighted rapid acquisition relaxation enhanced (RARE) sequence was performed with the following acquisition parameters: 29 axial slices, TE/TR: 23/1,784.52 ms, 256 × 256 image size, 14 × 14 field of view (FOV), and 0.7 mm slice thickness with 0.055 × 0.055 mm resolution. Single voxel (2.73 × 1.33 × 3 mm = 10 mm^3^) localized MR spectroscopy was performed using a Point RESolved Spectroscopy (PRESS) sequence with TE/TR: 18/2,500 ms, 64 averages and 0.81 Hz/points spectral resolution in the lymphoid nodules in the tongue. Data was analyzed using Paravision7.0 (Bruker) and LCModel (17). All lipid peaks were fitted using the LCModel and normalized to the lipid peak at 1.3 ppm (Lip 1.3). We choose to normalize to Lip 1.3 because it is the most abundant and stable peak among the lipid contents.

#### *In vivo* magnetic resonance imaging of the upper airway

*In vivo* MRI was performed on a subset of rats (*N* = 18) using a 7 T Bruker AVANCE III BioSpec MRI scanner (Bruker BioSpin Inc., Billerica, MA) and a four-element phased-array radiofrequency (RF) rat cardiac coil. Rats were anesthetized with 1.0–3.5% isoflurane (1–1.5 L/min in oxygen) via a nose cone, placed in ventral recumbency, and studied under eupneic conditions. A physiological monitoring and gating system (SA Instruments, Inc.) was used to monitor vital signs and for triggering respiratory-gated MRI scans. Body temperature was maintained at 36–37°C with warm air circulating in the magnet bore. T1-weighted MRI sagittal and axial scans of the upper airway lumen were performed with fat-suppression using a FLASH (Fast Low Angle Shot) sequence. Respiratory gating was used to trigger each scan to acquire images at the expiratory or inspiratory phase, respectively. Other imaging parameters included: 5 sagittal slices and 21 axial slices, slice thickness − 0.8–1.0 mm, in-plane resolution 111 × 156 μm, TR (repetition time) − 158.6 ms, TE (echo time) − 1.482 ms, and 6 averages. Image analysis and processing were performed using ParaVision 7 software (Bruker Biospin Corporation, 2020). Axial slices were used to measure the cross-sectional areas of the upper airway lumen. Both axial and sagittal slices were utilized for airway lumen cavity comparisons. Segmentation of the upper airway volume was performed using Segment (Medviso AB, Lund, Sweden), starting from one slice below the junction of the hard and soft palate to the slice at the root of the tongue.

#### Statistical analysis

Investigators involved with data collection were blinded to experimental group assignment. Data outliers for each variable were identified and re-checked for accuracy, but not removed from the dataset. The following dependent variables were assessed: (1) Plethysmography: frequency (f; breaths/min.), inspiratory time (TI; sec), expiratory time (TE; sec), tidal volume (VT/100 g; ml/100 g), minute ventilation (V.E/100 g; ml/min./100 g), mean inspiratory flow (VT/TI/100 g; ml/s/100 g), peak inspiratory flow (PIF; ml/s), peak expiratory flow (PEF; ml/s), and number of apneas (#); (2) MR spectroscopy: calculated lipid ratio (Lip2.1 + Lip2.3) normalized to the most abundant and stable peak among the lipid concentrations (Lip1.3); and (3) *in vivo* MRI of the upper airway: airway volume (mm^3^). Averaged values for each rat were used for plethysmography, and raw/unaveraged data were used for MR spectroscopy (i.e., single value per rat) and *in vivo* MRI of the upper airway. For plethysmography, separate two-way repeated measures ANOVAs were performed using SigmaPlot 15.0 (Systat Software, San Jose, CA) to compare the: (1) effect of time (repeated measure at baseline and endline), treatment groups (Control + sham exercise, Control + exercise, CTB-SAP + sham exercise, and CTB-SAP + exercise) and time×treatment group interactions on each outcome measure at each gas level (normoxia and max); and (2) effect of gas level (repeated measure at normoxia and max), treatment groups and level×treatment group interactions on each outcome measure at each timepoint (baseline and endline). For MR spectroscopy and *in vivo* MRI studies, a Students *t*-test was used to compare the relative concentration of Lip2.1 + Lip2.3 with respect to the Lip1.3 peak and differences in airway volume (mm^3^) between groups, respectively. If significant differences were indicated, multiple comparisons were made using a Fisher’s LSD *post hoc* test. Differences between groups were considered significant if *p* < 0.05, and all values were expressed as means ± 1 SEM.

## Results

### Intralingual CTB-SAP causes upper airway restriction that is decreased with tongue exercise

**Figure 1:**
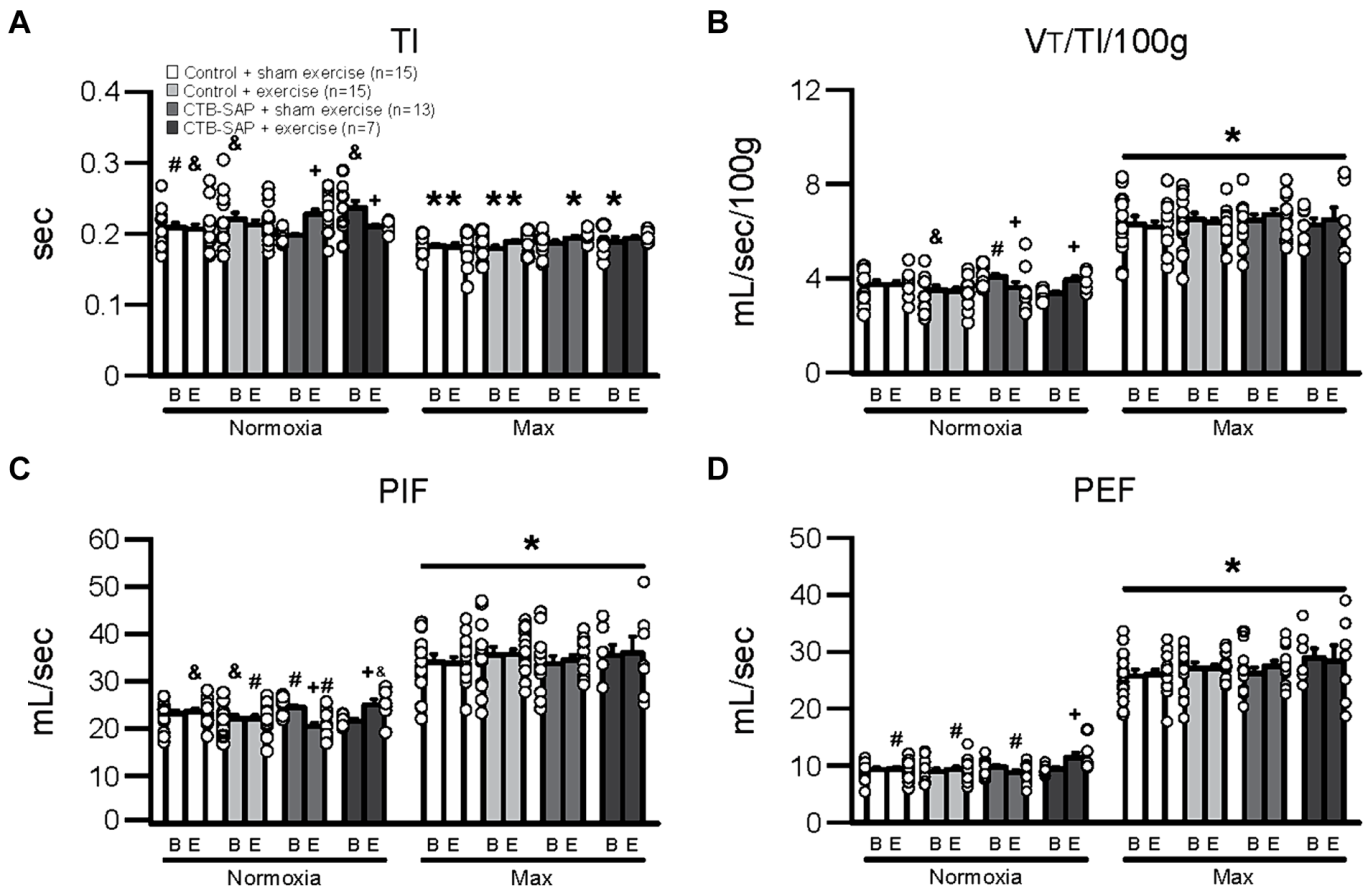
Breathing parameters [inspiratory time (TI), mean inspiratory flow (VT/TI/100 g), peak inspiratory flow (PIF), and peak expiratory flow (PEF)] at baseline (B) and endline (E) timepoints during normoxia and maximum chemoreceptor stimulation (max) for all groups (control + sham exercise, control + exercise, CTB-SAP + sham exercise, and CTB-SAP + exercise). All breathing parameters (TI, VT/TI/100 g, PIF and PEF) were significantly impacted when comparing max to normoxia during both baseline and endline timepoints (denoted by *). Some breathing parameters were significantly impacted in CTB-SAP + sham exercise rats vs. CTB-SAP + exercise rats (TI) and control + exercise rats (TI, VT/TI/100 g, and PIF) during normoxia at the baseline timepoint and vs. control + sham exercise rats (TI and PIF) and CTB-SAP + exercise rats (PIF) during normoxia at the endline timepoint (denoted by &). In addition, breathing parameters were significantly affected in CTB-SAP + exercise rats vs. control + sham exercise rats (TI) and CTB-SAP + sham exercise rats (VT/TI/100 g and PIF) during normoxia at the baseline timepoint and vs. CTB-SAP + sham exercise rats (PIF and PEF), control + exercise rats (PIF and PEF), and control + sham exercise rats (PEF) during normoxia at the endline timepoint (denoted by #). Lastly, breathing parameters (TI, VT/TI/100 g, PIF, and PEF) were significantly different when comparing baseline to endline timepoints during normoxia for CTB-SAP + sham exercise (TI, VT/TI/100 g, and PIF) and CTB-SAP + exercise groups (TI, VT/TI/100 g, PIF, and PEF; denoted by +). Values are expressed as means ±1 S.E.M., and differences were considered significant if *p* < 0.05. The adjacent dots to the left or right of each bar represent individual animal values.

Figure 1 depicts differences in breathing parameters [e.g., inspiratory time (TI), mean inspiratory flow (VT/TI/100 g), peak inspiratory flow (PIF), and peak expiratory flow (PEF)] for the four experimental groups at baseline and endline timepoints during normal breathing conditions (normoxia; 21% O_2_, balanced N_2_) and during max (hypercapnia + hypoxia; 7% CO_2_ + 10.5% O_2_, balanced N_2_). As hypothesized, all breathing parameters in Figure 1 were significantly decreased (TI) or increased (VT/TI/100 g, PIF and PEF) when comparing max to normoxia during both baseline [TI: control + sham exercise (*N* = 15; *p* = 0.003), control + exercise (*N* = 15; *p* < 0.001), and CTB-SAP + exercise (*N* = 7; *p* < 0.001); VT/TI/100 g: all experimental groups (*p* < 0.001); PIF: all experimental groups (*p* < 0.001); and PEF: all experimental groups (*p* < 0.001)] and endline timepoints [TI: control + sham exercise (*p* = 0.003), control + exercise (*p* = 0.009), and CTB-SAP + sham exercise (*N* = 13; *p* = 0.001); VT/TI/100 g: all experimental groups (*p* < 0.001); PIF: all experimental groups (*p* < 0.001); and PEF: all experimental groups (*p* < 0.001)]. As hypothesized, breathing parameters shown in Figure 1 were significantly different when comparing endline to baseline timepoints during normoxia for CTB-SAP + sham exercise (TI: increased, *p* < 0.001; VT/TI/100 g: decreased, *p* = 0.001; and PIF: decreased, *p* < 0.001) and CTB-SAP + exercise groups (TI: decreased, *p* = 0.013; VT/TI/100 g: increased, *p* = 0.010; PIF: increased, *p* = 0.002; and PEF: increased, *p* = 0.010). In addition, CTB-SAP + sham exercise rats had a significantly decreased TI vs. CTB-SAP + exercise rats (*p* = 0.004) and control + exercise rats (*p* = 0.032), and significantly increased VT/TI/100 g and PIF vs. CTB-SAP + exercise rats (VT/TI/100 g, *p* = 0.017; PIF, *p* = 0.026) and control + exercise rats (VT/TI/100 g, *p* = 0.018; PIF, *p* = 0.022) during normoxia at the baseline timepoint. In comparison, CTB-SAP + exercise rats had a significantly increased TI vs. control + sham exercise rats (*p* = 0.008) during normoxia at the baseline timepoint. During normoxia at the endline timepoint, CTB-SAP + sham exercise rats had a significantly increased TI vs. control + sham exercise rats (*p* = 0.029), a significantly decreased PIF vs. control + sham exercise rats (*p* = 0.001) and CTB-SAP + exercise rats (*p* < 0.001), and a significantly decreased PEF vs. CTB-SAP + exercise rats (*p* = 0.001). In comparison, CTB-SAP + exercise rats had a significantly increased PIF vs. control + exercise rats (*p* = 0.017) and increased PEF vs. control + exercise rats (*p* = 0.010) and control + sham exercise rats (*p* = 0.009) during normoxia at the end endline timepoint. There were no significant differences between experimental groups during max at the baseline or endline timepoints for TI, VT/TI/100 g, PIF, and PEF.

Summarized in Table 1 are the remaining outcome measures [e.g., frequency, expiratory time (TE), tidal volume (VT/100 g), and minute ventilation (V. E/100 g)] between the four experimental groups. Similar to the outcome measures in Figure 1, all breathing parameters were significantly increased (frequency, VT/100 g, and V.E/100 g) or decreased (TE) as hypothesized for all experimental groups when comparing max to normoxia during both baseline and endline timepoints (all *p* < 0.001). Only V.E/100 g was significantly different when comparing endline to baseline timepoints during normoxia for the CTB-SAP + sham exercise group (decreased, *p* = 0.026), while only VT/100 g was significantly different when comparing endline to baseline during max for the CTB-SAP + sham exercise group (increased, *p* = 0.019). There were no significant differences between experimental groups during normoxia at the baseline timepoint, and only V.E/100 g was significantly increased in CTB-SAP + exercise rats vs. control + exercise rats (*p* = 0.046) during normoxia at the endline timepoint but this small difference (64.7 ± 4.6 vs. 56.1 ± 2.4) is likely not biologically significant. During max at the baseline timepoint, CTB-SAP + sham exercise rats had a significantly decreased frequency vs. control + exercise rats (*p* = 0.011). In addition, during max at the endline timepoint, CTB-SAP + sham exercise rats had a significantly decreased frequency vs. control + sham exercise rats (*p* = 0.001) and increased VT/100 g vs. control + sham exercise rats (*p* < 0.001) and control + exercise rats (*p* = 0.048); CTB-SAP + exercise rats had a significantly decreased frequency (*p* = 0.012) and increased VT/100 g (*p* = 0.020) vs. control + sham exercise rats; and control + exercise rats had a significantly decreased frequency vs. control + sham exercise rats (*p* = 0.034). Lastly, the total number of apneas detected are summarized in Table 2. Total number of apneas were significantly decreased for all experimental groups when comparing max to normoxia during both baseline and endline timepoints (Table 2, all *p* < 0.001). Additionally, CTB-SAP + sham exercise treated rats had a significantly decreased number of total apneas vs. control + exercise rats (*p* = 0.044) during normoxia at the endline timepoint.

**Table 1:** Breathing parameters [frequency, expiratory time (TE), tidal volume (VT/100 g), and minute ventilation (V˙E/100 g)], at baseline and endline timepoints during normoxia and maximum chemoreceptor stimulation (max) for all groups (control + sham exercise, control + exercise, CTB-SAP + sham exercise, and CTB-SAP + exercise).

	Frequency (breaths/min)		TE (sec)		VT/100 g (ml/100 g)		V˙E/100 g (ml/min/100 g)	
Experimental Groups	Normoxia	Max	Normoxia	Max	Normoxia	Max	Normoxia	Max
Baseline								
Control + sham exercise	83.1 ± 3.1	159 ± 3.6*	0.56 ± 0.02	0.21 ± 0.00*	0.77 ± 0.03	1.14 ± 0.06*	61.2 ± 2.4	178 ± 9.0*
Control + exercise	78.0 ± 3.5	165 ± 4.2*^&^	0.59 ± 0.02	0.20 ± 0.00*	0.76 ± 0.04	1.16 ± 0.05*	57.6 ± 3.0	183 ± 7.3*
CTB-SAP + sham exercise	80.0 ± 2.6	151 ± 3.0*	0.59 ± 0.03	0.22 ± 0.00*	0.81 ± 0.03	1.20 ± 0.04*	62.8 ± 1.9	179 ± 6.3*
CTB-SAP + exercise	75.8 ± 5.3	157 ± 9.8*	0.61 ± 0.05	0.21 ± 0.01*	0.79 ± 0.03	1.19 ± 0.09*	57.9 ± 2.6	180 ± 8.3*
Endline								
Control + sham exercise	82.5 ± 3.2	165 ± 6.8*^&^^#^^^^	0.56 ± 0.03	0.20 ± 0.01*	0.76 ± 0.02	1.11 ± 0.04*^&^^#^	61.0 ± 2.3	175 ± 6.4*
Control + exercise	80.7 ± 2.6	153 ± 3.1*	0.58 ± 0.02	0.21 ± 0.00*	0.73 ± 0.03	1.20 ± 0.03*^&^	56.1 ± 2.4^#^	180 ± 4.2*
CTB-SAP + sham exercise	75.5 ± 3.5	146 ± 1.9*	0.61 ± 0.03	0.22 ± 0.00*	0.77 ± 0.02	1.30 ± 0.04*^+^	57.2 ± 2.5^+^	189 ± 5.9*
CTB-SAP + exercise	80.6 ± 3.4	148 ± 3.9*	0.58 ± 0.04	0.22 ± 0.01*	0.83 ± 0.04	1.25 ± 0.10*	64.7 ± 4.6	183 ± 16*

All breathing parameters (frequency, TE, VT/100 g, and V.E/100 g) were significantly impacted when comparing max to normoxia during both baseline and endline timepoints (denoted by *). Some breathing parameters were significantly impacted in CTB-SAP + sham exercise rats vs. control + exercise rats (frequency) during max at the baseline timepoint and vs. control + sham exercise rats (frequency and VT/100 g) and control + exercise rats (VT/100 g) during max at the endline timepoint (denoted by &). Breathing parameters were significantly affected in CTB-SAP + exercise rats vs. control + exercise rats (V.E/100 g) during normoxia at the endline timepoint and vs. control + sham exercise rats (frequency and VT/100 g) during max at the endline timepoint (denoted by #). Additionally, frequency was significantly different in control + exercise rats vs. control + sham exercise rats at max during the endline timepoint (denoted by ^). Lastly, breathing parameters were significantly impacted when comparing baseline to endline timepoints for the CTB-SAP + sham exercise group during normoxia (V.E/100 g) and during max (VT/100 g; denoted by +). Values are expressed as means ± 1 S.E.M., and differences were considered significant if *p* < 0.05.

**Table 2:** Apnea detection (total apneas) at baseline and endline timepoints during normoxia and maximum chemoreceptor stimulation (max) for all groups (control + sham exercise, control + exercise, CTB-SAP + sham exercise, and CTB-SAP + exercise).

	Total Apneas (#)	
Experimental Groups	Normoxia	Max
Baseline		
Control + sham exercise	12.9 ± 0.9*	0
Control + exercise	13.1 ± 1.6*	0
CTB-SAP + sham exercise	12.2 ± 0.8*	0.08 ± 0.1
CTB-SAP + exercise	13.9 ± 1.5*	0
Endline		
Control + sham exercise	11.8 ± 0.9*	0
Control + exercise	13.0 ± 1.1*^&^	0
CTB-SAP + sham exercise	10.5 ± 1.58*	0
CTB-SAP + exercise	10.9 ± 1.7*	0.14 ± 0.1

Number of total apneas were significantly impacted when comparing max to normoxia during both baseline and endline timepoints (denoted by *). Total apneas were significantly decreased in CTB-SAP + sham exercise rats vs. control + exercise rats during normoxia at the endline timepoint (denoted by &). Values are expressed as means ± 1 S.E.M., and differences were considered significant if *p* < 0.05.

### Increased lipid profiles in the tongue in CTB-SAP rats are mitigated by tongue exercise

**Figure 2:**
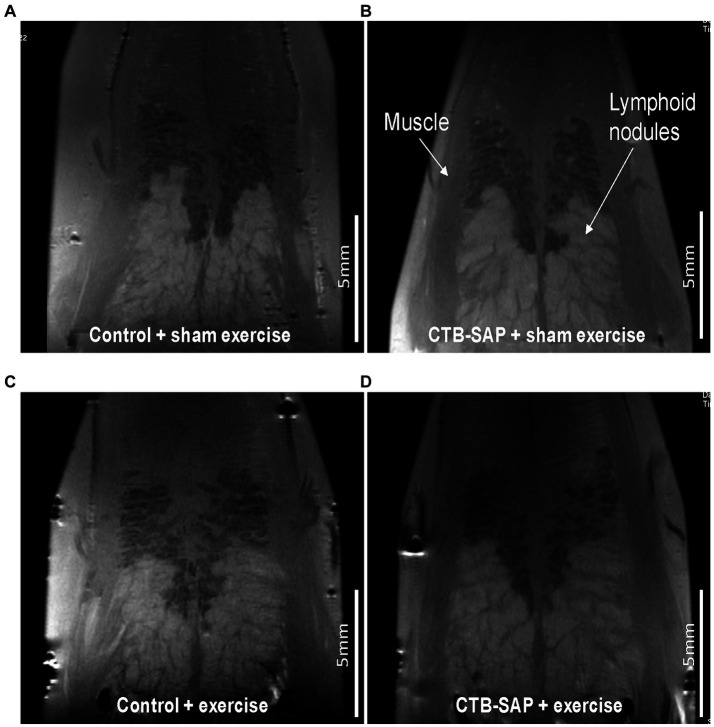
*Ex vivo* T2 weighted MRI of the tongue. Tongues from control + sham exercise **(A)**, control + exercise **(C)**, CTB-SAP + sham exercise **(B)**, and CTB-SAP + exercise treated rats **(D)**. Representative coronal slices are shown with lymphoid nodules and muscle indicated with white arrows **(B)**. Lymphoid nodules in the tongue in CTB-SAP + sham exercise rats **(B)** appear more extensive vs. control + sham exercise rats **(A)**.

**Figure 3:**
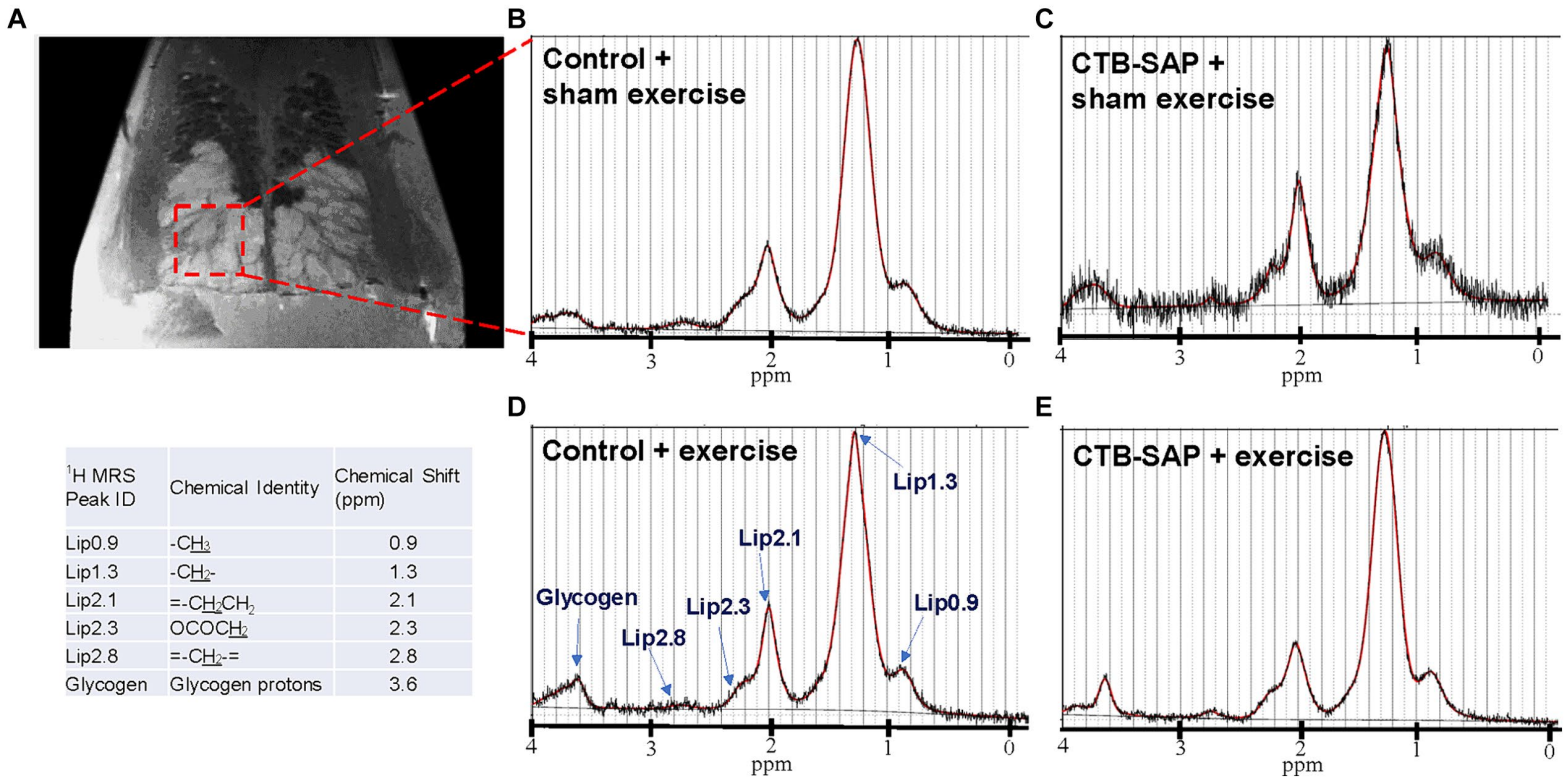
*Ex vivo* magnetic resonance spectroscopy (MRS) of the tongue. MRS of the lymphoid nodules in the tongue was performed in a single voxel (2.73 × 1.33 × 3 mm = 10 mm^3^), indicated by red dashed box on T2 weighted MRI **(A)**. Acquired MRS spectrum (black traces) and LC Model fitted lipid peaks (red traces) for each treatment group [control + sham exercise **(B)**, control + exercise **(D)**, CTB-SAP + sham exercise **(A,C)**, and CTB-SAP + exercise rats **(E)**] are shown. The major lipid peaks with their chemical shifts are indicated in **D** and the provided table, respectively.

**Figure 4:**
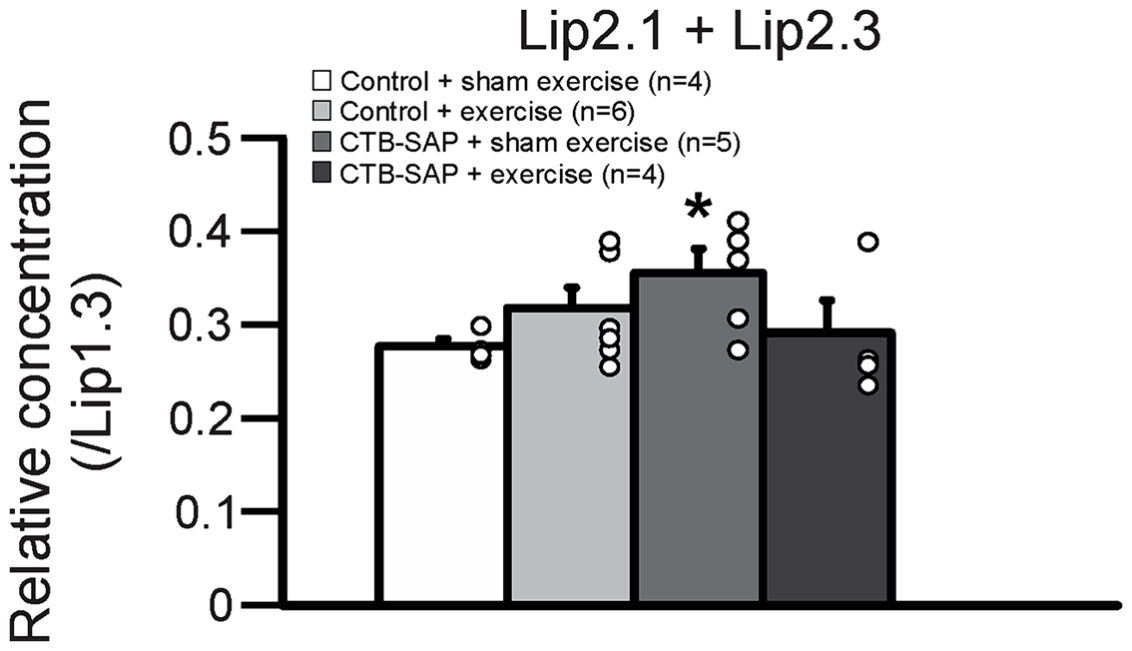
Relative lipid composition of the tongue lymphatic nodule tissue for all groups (control + sham exercise, control + exercise, CTB-SAP + sham exercise, and CTB-SAP + exercise). The relative concentration of Lip2.1 + Lip2.3 with respect to the Lip1.3 peak was significantly higher in CTB-SAP + sham exercise rats vs. control + sham exercise rats (denoted by *, *p* < 0.05). Values are expressed as means ±1 S.E.M. The adjacent dots to the right of each bar represent individual animal values.

*Ex vivo* T2-weighted (T2W) MRI was performed on the rat tongues to study the ultrafine structures of the tongue tissues and the lipid profiles of the lymphoid nodules in the tongue. The lymphoid nodules in the tongue showed hyper-intense signals as opposed to the tongue muscle on T2W MRI (Figure 2). Further, Figure 2 depicts the larger dimensions of the lymphoid nodules in the CTB-SAP + sham exercise rats as compared to the control + sham exercise rats. Further studies of the lymphoid nodules were carried out by MRS in a single voxel size (2.73 × 1.33 × 3 mm = 10 mm^3^). The lipid profiles were shown with the assigned lipid peaks and their respective chemical shifts in Figure 3. The spectra were fitted using LC Model software to obtain the relative concentrations of the lipid components. As shown in Figure 4, the ratio of Lip2.1 + Lip2.3 with respect to Lip1.3 was significantly higher in the CTB-SAP + sham exercise rats as compared to the control + sham exercise rats (*p* = 0.036). The CTB-SAP + exercise rats showed a decreased trend in the ratio of Lip2.1 + Lip2.3 to Lip1.3 as compared to the CTB-SAP + sham exercise rats but was not significant (*p* = 0.17).

### Intralingual CTB-SAP appears to induce structural changes in the upper airway of CTB-SAP rats that are mitigated by tongue exercise

**Figure 5:**
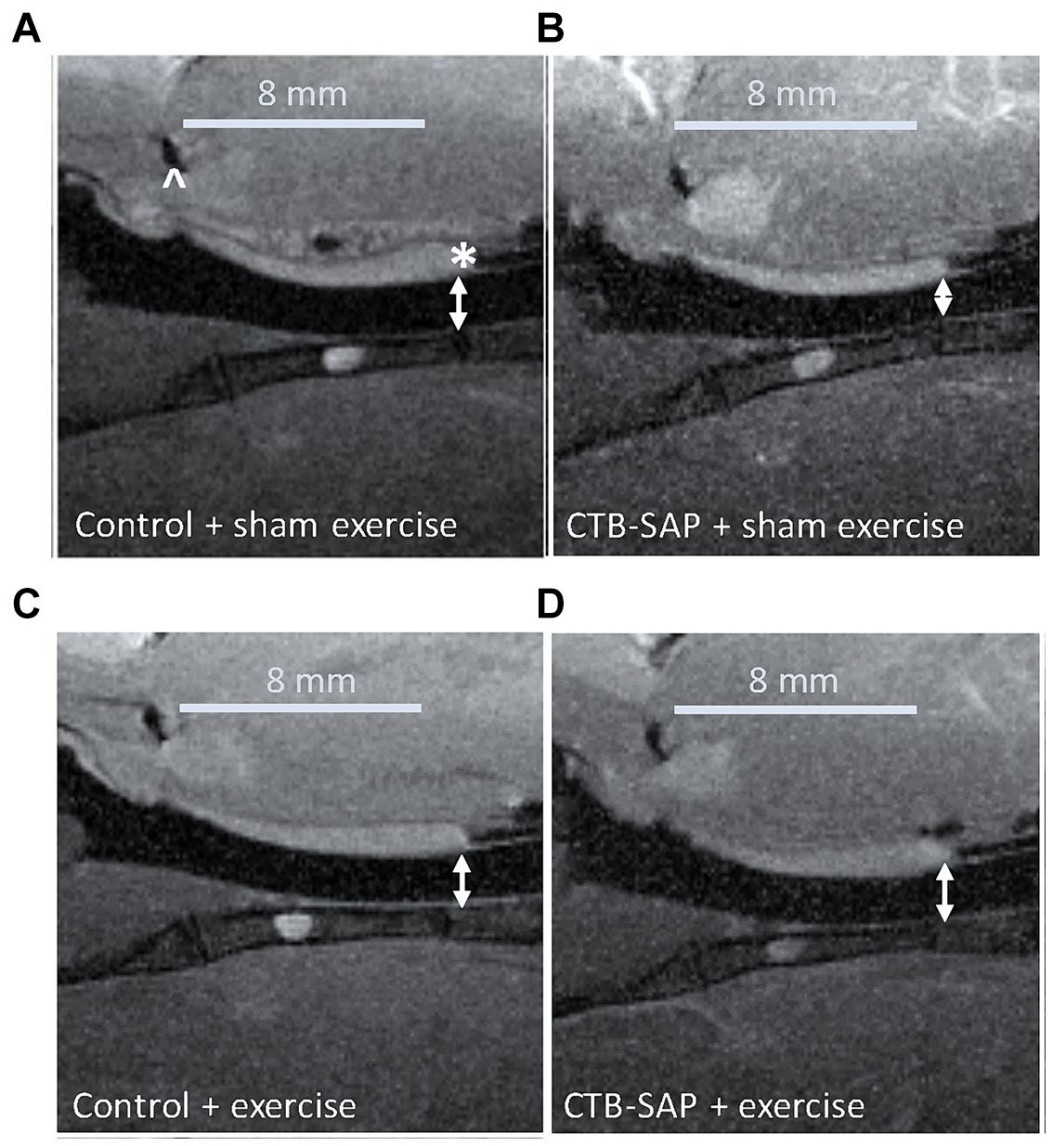
*In vivo* sagittal airway MRI. Airway volume (denoted by double-sided white arrows) evaluated from the junction of the hard and soft palate (denoted by a white * in **A**) through the slice representing the tongue root (denoted by a white ^ in **A**). The CTB-SAP + sham exercise treated rat **(B)** appears to show evidence of a compressed airway when compared to rats from the other treatment groups [i.e., control + sham exercise **(A)**, control + exercise **(C)**, and CTB-SAP + exercise rats **(D)**].

*In vivo* airway MRI (Figure 5) suggests that upper airway volume is significantly decreased in CTB-SAP + sham exercise rats (37.4 ± 3.1 mm^3^; *n* = 4) as compared to control + sham exercise rats (47.0 ± 2.2 mm^3^; *n* = 4; *p* = 0.04). However, after exercise (*n* = 5) we observed a significant expansion in upper airway volume for CTB-SAP rats (47.2 ± 0.3 mm^3^; *n* = 5), as compared to the CTB-SAP + sham exercise rats (*p* = 0.009), which is in close proximity to the average volume for control + sham exercise and control + exercise rat rats (47.4 ± 1.0 mm^3^; *n* = 5).

## Discussion

The main findings from this study were that: (1) There is evidence of upper airway restriction (i.e., reduced peak inspiratory flow and increased inspiratory time) in sham exercise-treated CTB-SAP rats vs. controls when studying breathing under normoxic conditions; (2) tongue exercise mitigated airflow deficits in CTB-SAP rats during normoxic conditions; (3) sham exercise-treated CTB-SAP rats have increased lipid expression in the tongue via *ex vivo* MR spectroscopy, consistent with previously observed tongue hypertrophy (15); (4) structural changes were present in the upper airway in sham exercise-treated CTB-SAP rats via *in vivo* MRI; and (5) strength endurance tongue exercise preserves upper airway structure and ultrafine structures in the tongue in CTB-SAP rats. Our previous studies have shown that ~60% of the hypoglossal motor neuron pool is lost due to intralingual injection of CTB-SAP, which results in hypoglossal motor deficits such as decreased motor output and motor neuron survival similar to what is seen in ALS mouse models (16, 18). This study provides novel evidence consistent with our hypothesis that tongue weakness in CTB-SAP treated rats results in restriction of the upper airway (e.g., decreased flow and increased inspiratory time) that can be mitigated with strength endurance tongue exercise, providing further evidence that it may be a viable therapeutic treatment for patients with hypoglossal-tongue axis degeneration in motor neuron diseases such as ALS and progressive bulbar palsy. Additionally, these studies reveal that lipid expression, as well as changes in upper airway diameter, are consistent with our previous findings of tongue hypertrophy (i.e., increased tongue volume and thickness, and marked hyperintensity of the tongue) due to hypoglossal motor neuron degeneration via intralingual injection of CTB-SAP (14).

### Tongue exercise has a beneficial therapeutic effect on respiratory function

We hypothesized CTB-SAP rats would show evidence of upper airway restriction compared to control rats during normoxic conditions. Here, CTB-SAP sham exercise-treated rats had significantly decreased indicators of airflow (i.e., peak inspiratory flow, peak expiratory flow, and mean inspiratory flow; surrogates for measuring potential changes in upper airway restriction) and significantly increased inspiratory time compared to CTB-SAP exercise-treated rats and control rats +/− exercise during normoxic conditions (Figure 1). These data provide further evidence that our strength endurance tongue exercise paradigm may restore airflow or prevent airflow deficits in CTB-SAP treated rats back towards control levels by preventing the characteristic weakened state of the tongue and limited tongue motion caused by degeneration, as observed in MNDs such as ALS (1).

As hypothesized, we did not anticipate evidence of upper airway restriction in CTB-SAP sham exercise-treated rats when challenged with hypercapnic + hypoxic conditions (10.5% O_2_ + 7% CO_2_; Figure 1; Tables 1, 2), providing evidence that CTB-SAP sham exercise-treated rats are somehow able to compensate and increase their respiratory function when challenged. This is consistent with studies involving *in vivo* neurophysiology utilizing intermittent or sustained hypoxia, which have shown evidence of respiratory plasticity known as hypoglossal long term facilitation (XII LTF) as a potential mechanism to increase upper airway tone and preserve upper airway patency (19–21). Additionally, this upper airway compensation could be due to the coactivation of muscles that work to protrude (e.g., genioglossus muscle), and retract (e.g., styloglossus and hyoglossus muscles) the tongue in response to a hypoxic + hypercapnic challenge (22, 23). However, whether XII LTF and/or muscle coactivation occurs during a hypoxic + hypercapnic challenge following CTB-SAP induced XII motor neuron loss remains unknown.

### *Ex vivo* MRI and MR spectroscopy of the tongue reveals altered lipid metabolism in CTB-SAP rats

Tongue fat is increased in patients with obstructive sleep apnea, suggesting that there are regional differences in fat distribution at the base of the tongue in apneic vs. non-apneic patients, which may affect the ability of the genioglossus to properly position the tongue away from the airway, causing restriction and loss of airway patency (24). Our previous *in vivo* MRI studies in this model revealed evidence of macrostructural changes in the tongue (e.g., significantly increased tongue volume and thickness, and marked hyperintensity of the tongue) consistent with potential muscle fiber inflammation, fatty replacement (possibly due to increased lipid concentration) of atrophied muscle fibers, +/− edema, which were somewhat mitigated via tongue exercise in CTB-SAP rats (15). Human ALS studies utilizing *in vivo* MRI reveal conflicting results as most studies have shown long-lasting disease characterized by decreased size (i.e., atrophic muscle fibers, increased connective tissue and fatty replacement) (25), while only a few describe enlarged tongues characterized as pseudohypertrophy due to denervation atrophy with fatty replacement caused by venous or lymphatic obstruction (26). Lipids have various roles, such as energy storage, membrane structure, and hormone synthesis. Due to metabolic disorders, inflammation, or injury, lipids accumulate in various tissues and can affect the function and viability of cells by changing their membrane properties, signaling pathways, and oxidative stress (27). Here, we showed an increase in the relative levels of the unsaturated fatty acid lipids (Lip2.1 + Lip2.3) to the saturated fatty acid (Lip1.3) in the rat tongue in CTB-SAP + sham exercise rats (Figures 2–4). The relative higher composition of the unsaturated fatty acid may indicate the activation of lymphatic macrophages in the regional tongue tissue. Importantly, macrophages can be pro-inflammatory in nature, secreting inflammatory cytokines such as IL-1β or IL-6 leading to host tissue damage (28), or anti-inflammatory in nature, secreting BDNF and supporting tissue remodeling (27). The role these potential lymphatic macrophages may play in regional tongue tissue of CTB-SAP rats is currently unknown but may contribute to inflammation and concurrent upper airway restriction. Future directions will focus on investigating the underlying mechanism responsible for tongue exercise-induced plasticity in the hypoglossal-tongue axis, particularly inflammatory associated factors such as BDNF.

### Tongue exercise preserves upper airway patency in CTB-SAP rats

An important factor that influences upper airway resistance is the diameter of the upper airway (29), and patients with MNDs such as ALS often have decreased muscle tone and strength in their upper airway that results in reduction of the airway lumen (30). We hypothesized CTB-SAP sham exercise-treated rats would have degenerative changes in their upper airway (i.e., compressed airway lumen) via *in vivo* MRI consistent with our plethysmography evidence of reduced airflow compared to CTB-SAP exercise-treated rats and control rats +/− tongue exercise. Our MRI results revealed significant upper airway constriction (i.e., decreased volume, mm^3^) in the oropharynx between the base of the tongue and the junction of the hard and soft palate in CTB-SAP + sham exercise-treated rats vs. control rats +/− tongue exercise, and CTB-SAP exercise-treated rats, suggesting that degenerative changes appear to be prevented by tongue exercise in CTB-SAP exercise-treated rats (Figure 5). Furthermore, our data are consistent with small changes in airway diameter leading to drastic changes in flow rate (29). Collectively, these findings suggest that a high-repetition/low endurance tongue exercise program could be used as an effective therapeutic to maintain upper airway patency in CTB-SAP rats.

### Limitations

Our studies involving *in vivo* MRI of the upper airway reveal a significant upper airway constriction in CTB-SAP + sham exercise rats (Figure 5). However, due to the narrow time window (less than 100 ms) that *in vivo* MRI images were taken, we were not consistently able to capture high quality images of the oropharynx at peak inspiration. Thus, our measurements of upper airway volume (mm^3^) may not consistently be representative of the upper airway at its peak capacity. The current *in vivo* MRI was performed using a heart imaging coil, which was not optimal for imaging the oropharynx. We expect to obtain higher quality images of the oropharynx at peak inspiration in future studies by using a new dedicated rat brain/neck RF coil and/or a higher field strength MRI, such as 9.4 T.

### Significance

Tongue exercise as a therapeutic option for patients with MNDs remains highly controversial in the absence of high rigor investigations (31–34). Low-repetition/high-resistance tongue exercise programs have been tested in a variety of rat models (e.g., primary aging, Parkinson’s disease, and ALS), where beneficial effects were shown for rat models of primary aging (35–37) and Parkinson’s disease (38, 39). However, an investigation using animal models of ALS concluded that tongue force training could result in detrimental effects on some measures of bulbar function (32), suggesting that low-repetition/high-resistance tongue exercise may prove to be a suitable therapeutic treatment for only some patients with ALS and other MNDs, but not in all cases. Thus, we chose to investigate a high-repetition/low-resistance exercise program that is tailored to prevent weakness, fatigue, and limited tongue movement caused by MNDs. Our current studies provide further evidence in support of high-repetition/low-endurance tongue exercise as a treatment option to preserve upper airway patency in patients with MNDs through improving airflow and preventing structural changes in the tongue and oropharynx. Therefore, we will continue leveraging our CTB-SAP rodent model to investigate the underlying mechanism of action responsible for tongue exercise-related treatment effects, and to optimize tongue exercise dosing parameters that will be translatable to humans. Further, these studies suggest MRI could offer a modality to detect and track degenerative/regenerative changes in the upper airway that could serve as a clinical tool to facilitate early diagnosis and treatment monitoring (and perhaps disease staging) in patients with MNDs such as ALS. MRI is widely available in human hospitals and non-invasive, providing an opportunity for earlier diagnosis and intervention when effective treatments become available.

## Data Availability

The raw data supporting the conclusions of this article will be made available by the authors, without undue reservation.
